# Downregulation of Thromboxane A_2_ Receptor Occurs Mainly via Nuclear Factor-KappaB Signaling Pathway in Rat Renal Artery

**DOI:** 10.1155/2017/6507048

**Published:** 2017-07-09

**Authors:** Yaping Zhang, Man Mi, Yan-Hua Xie, Si-Wang Wang, Lars Edvinsson, Cang-Bao Xu

**Affiliations:** ^1^Shaanxi Key Laboratory of Ischemic Cardiovascular Disease, Institute of Basic and Translational Medicine, Xi'an Medical University, Xi'an, China; ^2^Institute of Materia Medica, School of Pharmacy, Fourth Military Medical University, Xi'an, China; ^3^Division of Experimental Vascular Research, Institute of Clinical Science in Lund, Lund University, Lund, Sweden

## Abstract

Thromboxane A_2_ (TXA_2_) acts on TXA_2_ receptors (TP) to regulate renal artery blood flow and subsequently contributes to the pathogenesis of renal ischemia. The present study was designed to examine if nuclear factor-kappaB (NF-*κ*B) signaling pathway is involved in the downregulation of TP receptors in rat renal artery. Rat renal artery segments were organ cultured for 6 or 24 h. Downregulation of TP receptors was monitored using myograph (contractile function), real-time PCR (receptor mRNA), and immunohistochemistry (receptor protein). Specific inhibitors (MG-132 and BMS345541) for NF-*κ*B signaling pathway were used to dissect the underlying molecular mechanisms involved. Compared to fresh (noncultured) segments, organ culture of the renal artery segments for 24 h induced a significant rightward shift of U46619 (TP receptor agonist) contractile response curves (pEC_50_: 6.89 ± 0.06 versus 6.48 ± 0.04, *P* < 0.001). This decreased contractile response to U46619 was paralleled with decreased TP receptor mRNA and protein expressions in the renal artery smooth muscle cells. Specific inhibitors (MG-132 and BMS345541) for NF-*κ*B signaling pathway significantly abolished the decreased TP protein expression and receptor-mediated contractions. In conclusion, downregulation of TP receptors in the renal artery smooth muscle cells occurs mainly via the NF-*κ*B signaling pathway.

## 1. Introduction

Thromboxane A_2_ (TXA_2_) is mainly derived from platelets and vascular endothelial cells. It acts on TXA_2_ receptor (TP) to induce strong vasoconstriction in renal arteries [1, 2]. Increased renal vascular contractility and resistance can lead to reduced renal blood flow and subsequent renal ischemia [3, 4]. During experimental endotoxemic shock, renal vasoconstriction and ischemia were found to be mediated by TP receptors [3]. In mice with cell-specific deletion of TP receptors in vascular smooth muscle cells, TP receptor agonist U46619 failed to induce shock and hypertension, and angiotensin II-induced hypertension, vascular remodeling, and urinary thromboxane production were all significantly attenuated [5]. This indicated that TP receptors play a central role in the pathogenesis of these diseases. In endotoxemic mice, inhibition of cyclooxygenase (COX)-1 attenuated the formation of TXA_2_ which ameliorated the acute decrease in glomerular filtration rate [6]. Surgical reduction of renal mass in TP receptor knockout (TP-R−/−) mice demonstrated that activation of TP receptors mediated the increase in endothelin-1 (ET-1), reactive oxygen species (ROS), and microvascular remodeling and enhanced contractions in microvessels in chronic kidney disease [7], suggesting that TXA_2_ and its receptors were involved in the development of acute and chronic kidney injury.

On the other hand, TP receptor affinity was reduced and TP receptor-mediated vasoconstriction was decreased in gracilis muscle arterioles from pregnant rabbits [8] and in placentae from human diabetic pregnancies [9]. The phenomenon of decreased contractile response of vascular smooth muscle cells to TP receptor agonist U46619 was also seen in streptozotocin-induced diabetic rats [10]. Interestingly, renal production of TXA_2_ increased in diabetes [11], while TP receptor affinity and the receptor-mediated vasoconstriction decreased [9, 10]. Thus, the downregulation of TP receptors in renal vascular smooth muscle cells most likely serves as a protective mechanism to prevent renal ischemia. However, there is limited knowledge about the underlying molecular mechanisms responsible for downregulation of TP receptor expression.

Previously, we have established an organ culture model and demonstrated that organ culture of rat renal artery segments induced activation of nuclear factor-kappaB (NF-*κ*B) signaling transduction pathway and subsequently upregulated endothelin type A (ET_A_) and type B (ET_B_) receptors in the smooth muscle cells [12]. The present study was designed to investigate the underlying molecular mechanisms that are responsible for the downregulation of TP receptors in rat renal vascular smooth muscle cells.

## 2. Material and Methods

The present study followed previously published protocol by Xie et al. [12] to investigate the downregulation of TP receptors in rat renal artery.

### 2.1. Chemicals and Reagents

All chemicals were purchased from Sigma. U46619 was dissolved in ethanol to obtain a stock concentration of 10 mM and further diluted in distilled water. MG-132 and BMS345541 were dissolved in dimethyl sulfoxide (DMSO). Actinomycin D was dissolved in distilled water.

### 2.2. Tissue Preparation and Organ Culture Procedure

As previously described [12], male Sprague-Dawley rats (weighing 300–350 g) were anaesthetized with CO_2_ and exsanguinated. The renal artery was gently removed, immersed into cold buffer solution, and dissected free of adhering tissue under a microscope. The endothelium was denuded by perfusion of the vessel with 0.1% Triton X-100 for 10 s followed by another 10 s with a physiological buffer solution. The removal of the endothelium was verified by the absence of dilatory response to acetylcholine (10^−6^ M) in 5-hydroxytryptamine (10^−5^ M) precontracted segments. The vessels were cut into 1~1.5 mm long cylindrical segments, used directly (fresh group) or incubated at 37°C in humidified 5% CO_2_ in O_2_ for different time points (organ culture group). Segments for organ culture were placed in a 24-well plate, one segment in each well, immersed in 1 ml Dulbecco's modified Eagle's medium containing L-glutamine (584 mg/l), supplemented with penicillin (100 U/ml) and streptomycin (100 *μ*g/ml). For inhibition experiments, different inhibitors were added to the culture medium. The same volume of vehicle served as control. The experimental protocol was approved by the Lund University Animal Ethics Committee.

### 2.3. Buffer Solutions

Standard buffer solution (mM): NaCl 119; KCl 4.6; NaHCO_3_ 15; NaH_2_PO_4_ 1.2; MgCl_2_ 1.2; CaCl_2_ 1.5; glucose 5.5. In potassium-rich (60 mM K^+^) buffer solution, NaCl was replaced by an equimolar concentration of KCl in the standard solution. Analytical-grade chemicals and double-distilled water were used for the preparation of all solutions.

### 2.4. In Vitro Pharmacology

Fresh or incubated segments were immersed in a temperature-controlled (37°C) myograph system (Organ Bath Model 700MO, J.P. Trading, Aarhus, Denmark) containing 5 ml bicarbonate buffer solution. The solution was continuously aerated with 5% CO_2_ in O_2_ resulting in a pH of 7.4. The renal arterial segments were mounted for continuous recording of isometric tension by the Chart software (AD Instruments, Hastings, UK). Before exposure to potassium-rich buffer solution, a resting tone of 2.0 mN was applied to each segment and the segments were stabilized at this tension for 1.5 h. Resting tone at 2.0 mN is optimal in terms of length/active tension relationship and performed as previously described [13]. The potassium-induced contraction was used as a reference for the contractile capacity, and the segments were used only if potassium elicited reproducible responses over 1.0 mN. Concentration-response curves of U46619 were obtained by cumulative administration of the reagents, as previously described [12].

### 2.5. Real-Time RT-PCR

Total RNA extracted from fresh or cultured segments was reversely transcribed to cDNA. Real-time PCR was performed in a GeneAmp 7300 Sequence Detection system (Perkin-Elmer, Applied Biosystems), using the GeneAmp SYBR® Green kit (Perkin-Elmer, Applied Biosystems) with a 25 *μ*l reaction volume. The PCR reaction started at a temperature of 50°C for 2 min, then 95°C for 10 min, followed by 40 PCR cycles with 95°C for 15 s and 60°C for 1 min. Dissociation curves were obtained after the PCR cycles to identify the specific PCR products. *β*-Actin was used as housekeeping gene. The gene expressions were normalized against the housekeeping genes to account for differences in the starting material and in the cDNA reaction efficiency. The system automatically monitors the binding of a fluorescent dye to double-strand DNA by real-time detection of the fluorescence during each cycle of PCR amplification. Data were analyzed with the comparative cycle threshold (C_T_) method.

All primers were designed with the Primer Express 2.0 software (PE Applied Biosystems, CA, USA) and synthesized by TAG Copenhagen A/S (Copenhagen, Denmark). The nucleotide sequences of the primers used are shown in Table 1.

### 2.6. Immunohistochemistry

Fresh or cultured arterial segments were immersed in a fixative solution consisting of 4% paraformaldehyde in 0.1 M phosphate buffer (pH 7.4) at 4°C for 3 h. After fixation, the specimens were dehydrated at 4°C in phosphate buffer (0.1 M, pH 7.4) containing 20% sucrose for 24 h and then frozen in Tissue-Tek (Sakura Finetek Europe B.V., Zoeterwoude, The Netherlands) and stored at −80°C. Sections were cut into 10 *μ*m thickness in a cryostat and mounted on SuperFrost Plus slides. The sections were incubated overnight with rabbit anti-human TP (Cayman, 10004452, 1 : 200) in PBS with 10% fetal calf serum. The secondary antibodies were goat-anti-rabbit IgG FITC conjugated (Santa Cruz, sc-2012, 1 : 200) diluted in PBS. In control sections, either the primary or the secondary antibody was omitted. Positive staining in the smooth muscle cells was observed under a confocal microscope (C1plus; Nikon Instruments Inc., Melville, NY, USA) and analyzed by the Image J software (http://rsb.info.nih.gov/ij).

### 2.7. Data Analysis

All data are expressed as mean values ± SEM. Unpaired Student's *t*-test or two-way ANOVA with Bonferroni post test was used when two sets of data were compared and one-way ANOVA with Dunnett's multiple comparison test was used for comparisons of more than 2 data sets. *N*-number refers to preparations from different animals. A *P* value less than 0.05 was considered to be statistically significant.

## 3. Results

### 3.1. Downregulation of TP Receptor mRNA Expression and Receptor-Mediated Contractions in Renal Artery

Previously, we have shown that organ culture per se increased vasocontractile responses to ET-1 [12] and noradrenaline [14]; that is, after organ culture, the concentration-response curves of ET-1 and noradrenaline shifted to the left, and the maximal contractions increased [12, 14].

**Figure 1:**
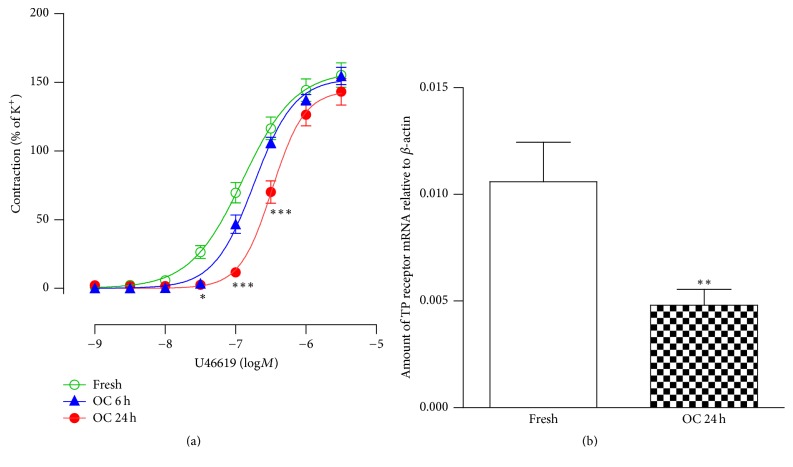
(a) Contractile responses to U46619 in the fresh segments or following organ culture for 6 or 24 h. Data are shown as means ± SEM (*n* = 8–16). Two-way ANOVA with Bonferroni post test was used. ^*∗*^*P* < 0.05,  ^*∗∗∗*^*P* < 0.001 versus fresh. (b) TP receptor mRNA expression in fresh renal arterial segments or organ cultured for 24 h. Data are shown as means ± SEM (*n* = 6–8). Unpaired Student's *t*-test was used. ^*∗∗*^*P* < 0.01 versus fresh. Fresh = noncultured segments; OC 6 h = organ cultured for 6 h; OC 24 h = organ cultured for 24 h.

The present study was designed to examine effects of organ culture per se on TP receptor expression. To study TP receptor mRNA expression and the receptor-mediated contractions, rat renal artery ring segments were cultured in the serum-free culture medium for 6 or 24 h. U46619 induced strong concentration-dependent contractions in fresh (noncultured) segments. Organ culture for 6 h made the contractile response curves of U46619 shift to the right, but the shift did not reach the statistical significance (pEC_50_: 6.89 ± 0.06 versus 6.73 ± 0.03, *P* > 0.05, Figure 1(a)) and there were no significant changes in the maximal contractions (*E*_max_: 158.2 ± 6.6 versus 152.9 ± 3.9, *P* > 0.05, Figure 1(a)). After 24 h of organ culture, there was a significant rightward shift in U46619 contractile response curves (pEC_50_: 6.89 ± 0.06 versus 6.48 ± 0.04, *P* < 0.001, Figure 1(a)), demonstrating downregulation of TP receptors in the renal artery smooth muscle cells.

K^+^-induced contraction was used as a reference of the contractile capacity. There was no significant difference in the K^+^-induced contractions before and after organ culture (before 1.62 ± 0.15 mN versus after 1.69 ± 0.22 mN, *n* = 14, *P* > 0.05).

**Figure 2:**
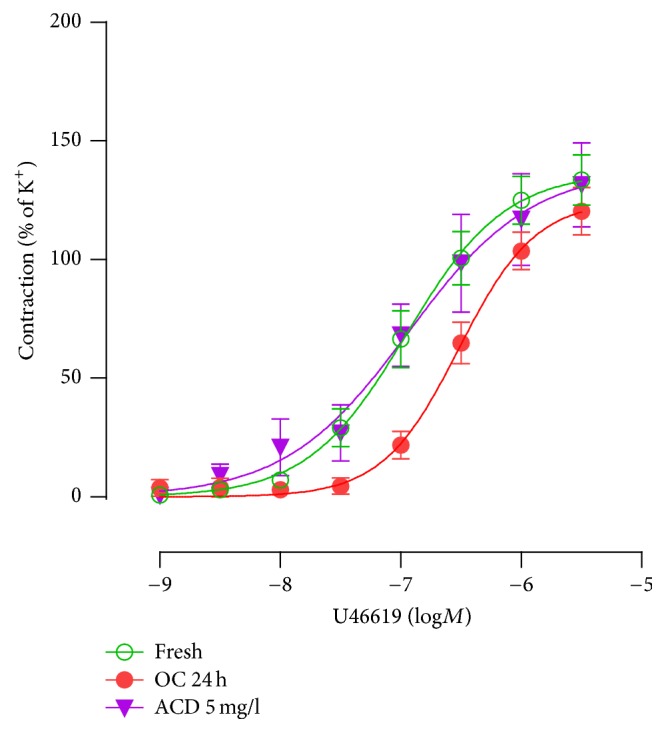
Effects of the transcriptional inhibitor actinomycin D (ACD 5 mg/L) on U46619-induced contraction. The artery segments were organ cultured in the presence of vehicle or ACD (5 mg/L) for 24 h. Fresh = noncultured segments. Data are shown as means ± SEM. Each data point is derived from 4–8 experiments.

The downregulation of TP receptor-mediated contractions was paralleled with significantly decreased TP receptor mRNA expression after 24 h of organ culture (Figure 1(b), *P* < 0.01), suggesting involvement of transcriptional mechanisms. This was further supported by the fact that a general transcriptional inhibitor actinomycin D (ACD 5 mg/L) normalized the downregulation of TP receptor-mediated contractions (Figure 2).

### 3.2. Inhibitors for NF-*κ*B Signaling Pathway Reversed the TP Receptor Downregulation

**Figure 3:**
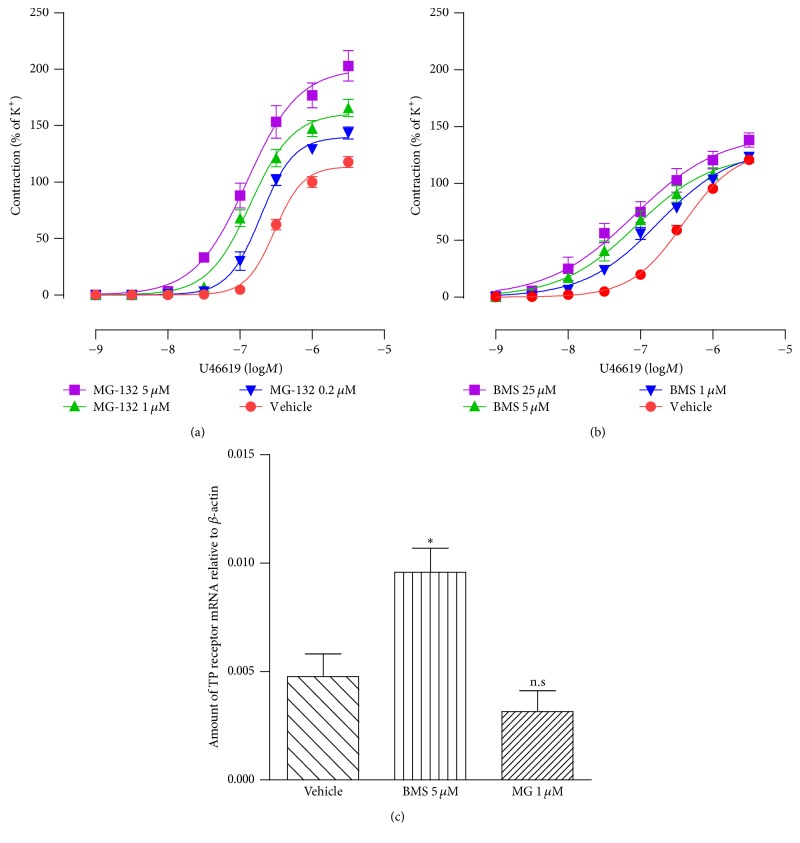
Effect of NF-*κ*B inhibitors (a) MG-132 and (b) BMS345541 on U46619-induced contraction and TP receptor (c) mRNA expression. Renal artery segments were organ cultured in presence of vehicle (DMSO) or different concentrations of MG-132 (MG, 0.2, 1, or 5 *μ*M) or BMS345541 (BMS, 1, 5, or 25 *μ*M) for 24 h. Data are shown as means ± SEM. Each data point is derived from 6 experiments. One-way ANOVA with Dunnett's multiple comparison test was used. ^*∗*^*P* < 0.05 versus vehicle (DMSO) and n.s. = not significant. BMS = BMS345541, MG = MG-132.

I*κ*B kinase (IKK) is part of the upstream NF-*κ*B signaling transduction cascade. It phosphorylates the inhibitory I*κ*B*α* protein and subsequently results in the dissociation of I*κ*B*α* from NF-*κ*B, allowing free NF-*κ*B to translocate into the nucleus to induce gene expressions. In our established organ culture model, only a faint expression of the phosphorylated IKK protein was demonstrated in the renal artery smooth muscle cells of fresh segments (before organ culture), while after organ culture, the expression of the phosphorylated IKK protein significantly increased, indicating that organ culture per se activated NF-*κ*B signaling transduction pathway [12]. In addition, Western blot experiments showed that organ culture per se time-dependently induced phosphorylation of NF-*κ*B in smooth muscle cells, and migration of NF-*κ*B from the cytoplasm to the nucleus [15]. In the present study, the same model was used with two specific NF-*κ*B signaling pathway inhibitors: MG-132 (0.2, 1, and 5 *μ*M), which is ubiquitin-proteasome inhibitor that inhibits NF-*κ*B nuclear translocation [16], and BMS345541 (1, 5, and 25 *μ*M), which is an I*κ*B inhibitor [17]. Both MG-132 (Figure 3(a), Table 2) and BMS345541 (Figure 3(b), Table 2) concentration-dependently reversed the downregulation of TP receptor-mediated contractions.

In addition, BMS345541 (5 *μ*M) significantly abolished the decreased TP receptor mRNA expression (Figure 3(c)). However, MG-132 (1 *μ*M) surprisingly did not affect TP receptor mRNA expression (Figure 3(c)).

### 3.3. Regulation of TP Receptor Protein Expression

**Figure 4:**
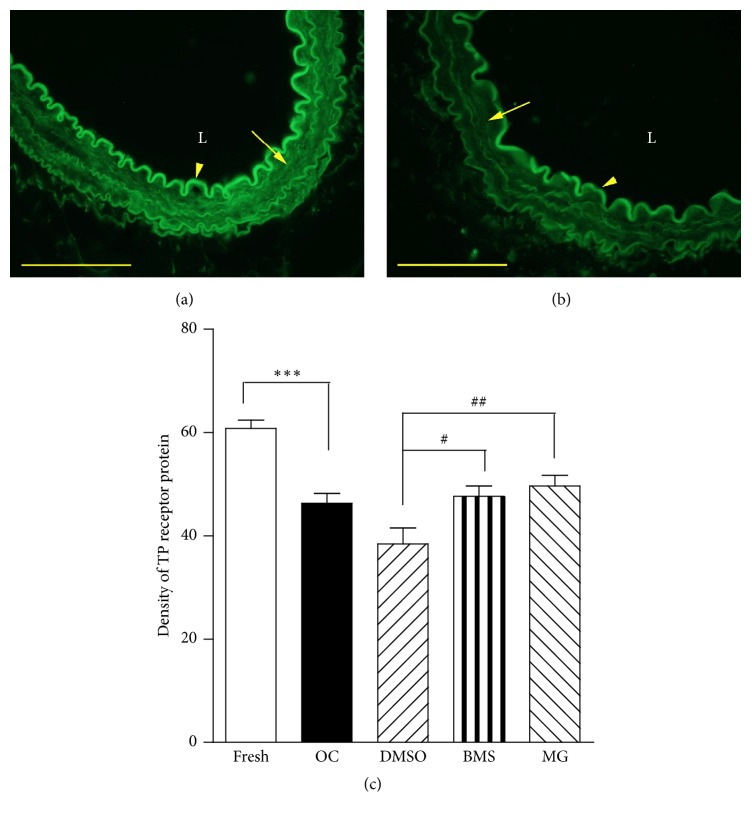
TP receptor protein expression in the fresh segments (a) and following organ culture for 24 h (b). The arteries were denuded of endothelium; the innermost autofluorescent inner elastic membrane is easily depicted (arrow head). The smooth muscle cell protein expression is homogenously distributed (arrow). L indicates lumen. The scale bar = 100 *μ*m. The protein expression was quantified using the program Image J software (c). Each data point is derived from 6 experiments. Unpaired Student's *t*-test was used. ^*∗∗∗*^*P* < 0.001 versus fresh; ^#^*P* < 0.05,  ^##^*P* < 0.01 versus vehicle.

TP receptor protein expression was investigated by immunohistochemistry in the renal artery segments after removal of the endothelium. Strong TP receptor protein immunoreaction (expression) with bright green granules located in the smooth muscle cells was seen in fresh controls (nonculture, Figure 4(a)). Organ culture for 24 h resulted in decreased TP receptor protein expression in the smooth muscle cells (Figure 4(b)). Semiquantitative measurement of the receptor protein densities using Image J software showed that inhibition of NF-*κ*B signaling pathway using MG-132 or BMS345541 significantly reversed the downregulation of TP receptor protein expressions (Figure 4(c)).

## 4. Discussion

Under physiological conditions, TP receptors play an important role in maintenance of renal blood flow, mediation of renin release, and regulation of sodium excretion [4]. During renal ischemia, TP receptor mediates renal artery contraction and contributes to increased kidney injury [3]. The present study has demonstrated that organ culture of renal artery segments induced downregulation of TP receptor expressions with decrease in receptor-mediated contraction in renal artery smooth muscle cells. Furthermore, the downregulation of TP receptor expressions occurred mainly via activation of NF-*κ*B signaling pathway. Our findings suggest that the organ culture method provides a model to study molecular mechanisms that are responsible for the regulation of TP receptor expressions. The downregulation of TP receptor expressions may contribute to modulation of renal artery blood flow and serve as a feedback mechanism in the pathogenesis of renal ischemia.

TXA_2_ acts on TP receptors in renal smooth muscle cells to induce renal vascular contraction. Infusion of angiotensin II at a slow pressor rate caused renal vasoconstriction and hypertension [18], which was prevented by deletion of the gene for TP receptors in the vascular smooth muscle cells [5], suggesting that the slow pressor dose of angiotensin II infusion could increase production of TXA_2_ and subsequently induce renal vasoconstriction [18, 19]. In the present study, organ culture of renal artery for 6 and 24 h resulted in time-dependent downregulation of TP receptor expression in the renal smooth muscle cells and subsequently decreased contractile response to U46619. A general transcription inhibitor actinomycin D normalized the downregulation of TP receptor-mediated contractions, suggesting involvement of transcriptional mechanisms. Inhibition of NF-*κ*B signaling pathway by using MG-132 and BMS345541 significantly abolished the downregulation of TP receptor-mediated contractions as well as the decrease in receptor mRNA and protein expressions in smooth muscle cells, suggesting that the downregulation of TP receptors occurred mainly via activation of NF-*κ*B signaling transduction pathway.

BMS345541 significantly abolished the decreased TP receptor mRNA expression, while MG-132 did not significantly affect TP receptor mRNA expression. This might be due to complex mechanisms involved in the regulation of TP receptor mRNA and protein expressions. In fact, not only is protein expression controlled by transcriptional mechanisms [20], but posttranscriptional, translational, and protein degradation mechanisms are also involved in the control of steady-state protein abundances [21]. Similar mRNA expression patterns could be accompanied by large variations (more than 20-fold difference) of protein abundances, and vice versa [22]. Thus, mRNA expression poorly predicts the corresponding protein expression level, and mRNA-protein expression correlation might also vary in different treatments.

Increased renal artery contractility leads to reduced renal blood flow and subsequent renal ischemia, which is often seen in renal vasospasm [23] and endotoxemic shock-induced renal injury [3]. On the other hand, downregulation of TP receptors occurs in pregnancy and diabetes [8–10]. In the present study, organ culture per se induced downregulation of TP receptors in rat renal smooth muscle cells. This simulates TP receptor downregulation which is a phenomenon seen in pregnancy and diabetes [8–10]. Thus, organ culture method may be a useful model for studying the underlying molecular mechanisms responsible for regulation of the TP receptor expression.

## 5. Conclusion

Organ culture simulates TP receptor downregulation, which is a phenomenon seen in pregnancy and diabetes [8–10]. Downregulation of TP receptors in renal artery smooth muscle cells mainly occurs via NF-*κ*B signaling transduction pathway-mediated transcriptional mechanisms and might contribute to modulation of renal artery blood flow and serve as a protective feedback mechanism to prevent renal ischemia and kidney injury.

## Figures and Tables

**Table 1:** Accession numbers and primer sequence for the genes that were investigated.

Gene name	Abbreviation	Accession number	Primer sequence
Thromboxane A_2_ receptor	TP	NM_017054.1	Fwd: 5′-ATCTCCCATCTTGCCATAGTCC-3′
			Rev: 5′-CCGATGATCCTTGAGCCTAAAG-3′
beta-Actin	ACTB	NM_031144.2	Fwd: 5′-GTAGCCATCCAGGCTGTGTTG-3′
			Rev: 5′-TGCCAGTGGTACGACCAGAG-3′

**Table 2:** Maximal contractile response (*E*_max_) and pEC_50_ values to U46619 in rat renal arteries following organ culture for 24 h in presence of vehicle (DMSO) or different concentration of NF-*κ*B inhibitors.

Groups	*n*	U46619	
		*E*_max_ (%)	pEC_50_
Vehicle	6	113.7 ± 2.30	6.52 ± 0.02
MG 0.2 *μ*M	6	140.1 ± 3.35^*∗*^	6.71 ± 0.03^*∗*^
MG 1 *μ*M	6	161.3 ± 5.23^*∗∗∗*^	6.86 ± 0.04^*∗∗∗*^
MG 5 *μ*M	6	200.6 ± 9.25^*∗∗∗*^	6.90 ± 0.07^*∗∗∗*^
vehicle	6	129.3 ± 4.19	6.41 ± 0.04
BMS 1 *μ*M	6	130.7 ± 5.52	6.75 ± 0.07
BMS 5 *μ*M	6	125.8 ± 7.49	7.06 ± 0.10^*∗∗*^
BMS 25 *μ*M	6	143.7 ± 11.68	7.09 ± 0.16^*∗∗∗*^

Overview of *E*_max_ and pEC_50_ values of U46619-induced contractile response on rat renal arteries. The segments were organ cultured for 24 h in presence of vehicle (control) or different concentrations of MG-132 (MG 0.2, 1, or 5 *μ*M) or BMS345541 (BMS 1, 5, or 25 *μ*M). Data are shown as mean ± SEM. *n* denotes the number of vessel segments. One-way ANOVA with Dunnett's multiple comparison test was used. ^*∗*^*P* < 0.05,  ^*∗∗*^*P* < 0.01,  ^*∗∗∗*^*P* < 0.001 versus vehicle.
